# Lights and Shadows of Gold Introduction into Beta Zeolite

**DOI:** 10.3390/molecules25245781

**Published:** 2020-12-08

**Authors:** Adrian Walkowiak, Lukasz Wolski, Maria Ziolek

**Affiliations:** Faculty of Chemistry, Adam Mickiewicz University, Poznań, Uniwersytetu Poznańskiego 8, 61-614 Poznań, Poland; wolski.lukasz@amu.edu.pl (L.W.); ziolek@amu.edu.pl (M.Z.)

**Keywords:** gold nanoparticles, wet impregnation, ion exchange, deposition-reduction, grafting with organosilane, efficiency of gold loading, zeolite acidity, zeolite porosity, zeolite desilication

## Abstract

Four different methods for gold deposition on Beta zeolite, namely impregnation, ion-exchange, deposition-reduction, and grafting on (3-aminopropyl)trimethoxysilane functionalized support, were applied to investigate their influence on textural/structural changes in the zeolite support and its surface acidity. The as-prepared materials were fully characterized by XRD, N_2_ physisorption, ICP-OES, XPS, TEM, and pyridine adsorption. The obtained results indicated that bifunctional redox–acidic materials prepared within this work were characterized not only by different gold loading and gold particle size, but also different textural parameters and acidity. All these features were strongly affected by the procedure applied for gold deposition. The introduction of Au into Beta zeolite by ion exchange caused a significant decrease in the Si/Al ratio in the zeolite framework. The size of Au particles determined the textural parameters of the zeolite and the number of Lewis acid sites (LAS). The Brønsted acid sites (BAS) number was decreased if (3-aminopropyl)trimethoxysilane or NaBH_4_ were used in the procedure of gold deposition. The highest BAS/LAS ratio was achieved for the sample prepared by ion exchange in the ammonium form of Beta zeolite. The presented results permit making a proper choice of the gold modification procedure for the preparation of bifunctional (redox–acidic) materials, addressed to a desired application.

## 1. Introduction

The era of gold catalysts started by the Haruta’s famous work [1] continues. Different supports for gold nanoparticles have been applied, among them carbons (e.g., [2,3]), metal oxides (e.g., [1,4]), silica, including ordered mesoporous silicas (e.g., [5,6]), and zeolites [7,8,9,10,11] which have been widely presented and discussed in literature over many years since the time of Haruta’s discovery of gold nanoparticles (NPs) catalytic activity. The supports can be inactive as silica or can have their own activity, like the other supports mentioned above. The active supports are important in the production of bifunctional catalysts useful in many catalytic reactions, e.g., in the oxidation of alcohols in which redox and acidic centers are involved [12,13,14,15,16,17]. Zeolites belong to the active supports in which the type (Lewis or Brønsted), concentration, and strength of acidic centers can be easily modified via, e.g., dealumination, cation exchange, or isomorphous substitution of different elements. Therefore, zeolites are of much interest in the formation of bifunctional catalysts containing gold as active redox centers.

Various aspects have been considered in the development of effective gold catalysts. One of them is the method of gold loading on the support. Most of the techniques applied for this purpose has been described in the recent review paper [18]. The authors have discussed the procedures, effectiveness of gold loading and sizes of gold NPs determined by the use of the following methods: deposition/precipitation (DP), co-precipitation (CP), impregnation (IM), vapor-phase deposition (VPD), grafting, sol-gel (SG) and ion-exchange (IE). The other methods proposed in literature include the deposition-reduction (DR) technique [19] and grafting on the supports functionalized with amino-organosilanes [20,21] or mercapto-organosilanes [14,22]. The most frequently used gold source for the deposition of Au on the supports is chloroauric acid (HAuCl_4_).

The idea of this work was to apply four different methods for gold NPs deposition on Beta zeolite: impregnation (IM), ion-exchange (IE), deposition-reduction (DR), and grafting on (3-aminopropyl)trimethoxysilane (APTMS) functionalized support. The choice of methods for Au loading on the support followed from the use of different compounds for zeolite treatment that would ensure gold anchoring and transformation to metallic species. The source of gold in all above-mentioned methods was chloroauric acid (tetrachloroaurate hydrate). In the methods of impregnation and ion-exchange, only aqueous solution of hydrogen chloroauric acid was used for zeolite treatment at different conditions depending on the method. The reduction of gold source in both techniques occurred during the calcination step. In the deposition-reduction method, sodium borohydride was used as a strong reducing agent. Thus, a zeolite was subject to changes resulting from the effect of this compound on the structure and surface properties of the support. In the fourth method, zeolite was treated with APTMS before the admission of gold precursor. This treatment could also change the surface properties of the support. The focus in this work was mainly on variations in zeolite properties caused by the application of different gold modification methods, i.e., the topic less investigated than the role of the Au loading method on gold NPs size and Au–support interaction. Thus, the idea is to show the lights and shadows of each method used for gold deposition with respect to shaping the properties of the support, zeolite.

The choice of Beta zeolite as a support for gold was caused by the commercial availability and unique properties of this molecular sieve. The structure of this large-pore zeolite was thoroughly investigated by Newsam et al. [23]. According to them, Beta zeolite can be viewed as a hybrid of at least two intergrown polymorphs (denoted as A and B) consisting of a three-dimensional pore system that is accessible through 12-membered rings [23,24,25]. Hence, Beta differs from the other zeolites by a large number of stacking defects. Owing to its high acidity, large external surface area, and quite good thermal resistance, it has been used in many industrial processes, such as the alkylation and acylation of arenes [26,27], as well as the cracking and isomerization of hydrocarbons [28,29,30].

## 2. Results and Discussion

### 2.1. Composition of Materials and Effectiveness of Gold Loading

As mentioned in Section 1, the choice of a method for introducing gold onto the support could have a crucial impact on the effectiveness of this metal loading. In this study, the efficiency of gold embedding with the use of four different techniques was compared. The amount of introduced gold was measured by ICP-OES after digestion of the sample in a mixture of acids. The results obtained are summarized in Table 1. It is worth emphasizing that the assumed gold loading in the case of all the samples was of 2 wt.%.

The highest efficiency of gold loading was achieved for the samples prepared by wet impregnation (Au-HBeta(IM)) and anchoring of gold species on the surface of the zeolite grafted with APTMS (Au-HBeta(AP)). The efficiency of Au loading in these cases reached about 70–75%. The high efficiency of Au introduction in Au-HBeta(AP) can be explained by a strong interaction between [AuCl_4_]^−^ ions with −NH_3_^+^ groups resulting from protonation of −NH_2_ modifier’s groups [20]. It is worth noting that the loading of gold on Beta zeolite by the deposition-reduction method (Au-HBeta(DR)) led to ca. twice lower efficiency than that observed for the samples prepared by wet impregnation and grafting.

It is undoubtedly worth noting that the efficiency of gold deposition onto zeolite Beta via ion exchange strictly depends on the form of zeolite to be modified (hydrogen—HBeta vs. ammonium—Beta). As implied by Table 1 data, the efficiency of Au loading was several times lower for the hydrogen form than for the ammonium form of the zeolite (see samples Au-HBeta(IE) and Au-Beta(IE)_18h, respectively). This phenomenon resulted from the fact that protons are more strongly bound to the zeolite framework than ammonium ions, which only electrostatically interact with the zeolite skeleton and are known for their high exchangeability. Similar results have been obtained by Sanada et al. [30] who documented that the efficiency of gold introduction by ion exchange method to the ammonium form of zeolite Y was much higher than that observed for the hydrogen form of the zeolite. The authors associated the excellent performance of this method with the formation of NH_4_Cl resulting from a combination of chloride anions originating from the coordination sphere of tetrachloroaurate anions and ammonium cations neutralizing the zeolite skeleton’s negative charge.

In order to estimate the impact of reaction time on ion exchange efficiency in the ammonium form of Beta, the synthesis duration was increased from 18 to 42 h. As implied by Table 1 data, the extended reaction time contributed to a slight increase in the efficiency of gold loading. For this reason, the sample obtained for a longer time of ion exchange was selected for further studies.

ICP-OES measurements were also carried out to determine the Si/Al molar ratio in the examined samples. The Si/Al ratio of commercial Beta declared by the supplier was 19. The value of Si/Al molar ratio for this material obtained from ICP-OES was slightly lower and was found to be 17.8. It is important to stress that Si/Al ratio estimated for almost all the samples was close to that observed for the parent zeolite (see Table 1). Nonetheless, the material prepared by the ion exchange in the NH_4_^+^-form of Beta zeolite with HAuCl_4_ (Au-Beta(IE)) exhibited a significantly lower Si/Al ratio than the other samples. It must be taken into account that, before the ion exchange procedure, the pH value of the gold precursor solution was adjusted to ~6.0 with the use of a sodium hydroxide solution. Literature reports [32,33,34,35,36] indicate that the addition of NaOH can result in conversion of [AuCl_4_]^−^ anions into [AuCl*_x_*(OH)_4−*x*_]^−^ (where *x* is an integer from 0 to 3; e.g., [AuCl_2_(OH)_2_]^−^, [AuCl(OH)_3_]^−^) and into non-ionic complexes [AuCl*_y_*(OH)_3−*y*_(H_2_O)] (where *y* is an integer from 0 to 3; e.g., [AuCl_3_(H_2_O)], [AuCl_2_(OH)(H_2_O)]). It has been established [37,38] that the gold zero-charged complexes play the major role in the exchange process. The following mechanism was proposed in [39]: AuX_2_(OH)_(aq)_ + H^+^Zeolite → [AuX_2_]^+^Zeolite + H_2_O, where X was OH^−^ or Cl^−^. In zeolites containing ammonium cations instead of protons this process proceeds even easier because interaction of NH_4_^+^ cations with the negatively charged zeolite framework is weaker than the interaction of protons. A similar mechanism describing such an interaction of the zero-charged gold species with a negatively charged titania surface was also postulated by Moreau et al. [38]. Literature data show [32] that hydroxide ions can be released from the above-mentioned gold complexes upon heat treatment. Since the ion-exchange was performed in our study at 80 °C for 42 h, we claimed that partial desilication of the zeolite matrix observed for Au-Beta(IE) sample was caused by hydroxide ions released from the gold complexes. For sample Au-HBeta(IE), for which Au loading was very low, no considerable decrease in silicon content was observed.

### 2.2. Structure/Texture Characterization

**Figure 1:**
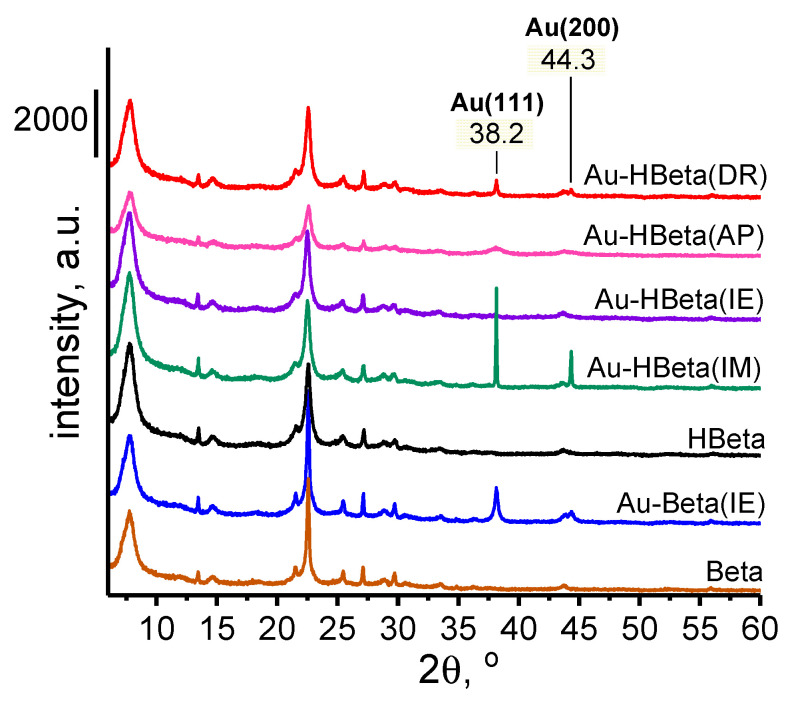
XRD patterns of zeolites. The peaks originating from metallic gold species were marked.

X-ray diffraction patterns for the parent Beta zeolite and the materials based on it, are shown in Figure 1. Diffraction peaks characteristic of the Beta zeolite structure appear in the range from 6 to 35° 2*θ*. The peaks at 7.6° 2*θ* and 14.7° 2*θ* are broadened due to the stacking faults in the structure of Beta zeolite resulting from intergrowth of polymorphs A and B. Such a phenomenon is an intrinsic feature of this material [40]. It should be noted that there are no differences in the peak positions for parent supports and gold containing zeolites. As follows from analysis of the XRD patterns recorded, no method of Au introduction applied in this work has brought about major changes in structural properties of the zeolite matrix.

For gold-containing samples, two additional diffraction peaks appear at 38.2° and 44.3° 2*θ*, which are assigned to X-ray reflections from the planes (111) and (200) of metallic gold particles, respectively [41]. On the basis of the former peak, the gold particle sizes were calculated, which will be discussed in more detail below. The presence of gold nanoparticles in the examined samples was also confirmed with the use of UV-Vis spectrophotometry. As shown in Supplementary Figure S3, after the introduction of gold, for all samples, an additional absorption band in the range from ca. 500 to 600 nm appeared. According to the literature, this absorption band is attributed to the surface plasmon resonance (SPR) phenomenon on Au nanoparticles [42,43].

Nitrogen adsorption-desorption isotherms for the examined materials are shown in Supplementary Figure S4. In accordance with International Union of Pure and Applied Chemistry (IUPAC) recommendations [44] the IV type of isotherm was assigned to all the samples. The hysteresis loops are of the H4 type and are typical of particles containing small pores, including micropores. They are interpreted as resulting from mesoporosity arising from aggregated zeolite grains [44]. Although the low-temperature adsorption of nitrogen is not seen as a reliable method for the examination of textural properties of microporous materials, such as zeolites [44], it has been widely used for this purpose.

As evidenced in Table 2, the total BET (Brunauer-Emmett-Teller) surface area of parent Beta zeolite was of 521 m^2^·g^−1^, which is in agreement with literature data [45,46]. The BET surface areas of hydrogen form of Beta zeolite (HBeta), Au-Beta(IE), Au-HBeta(IM), and Au-HBeta(IE) were found to be of 527, 516, 492, and 520 m^2^·g^−1^, respectively, and they differed only slightly from that of the parent zeolite support. This indicates that Au introduction had a negligible influence on the textural properties of the zeolite support. The exception was Au-HBeta(DR), whose BET surface area was decreased by about 10% in comparison to that of the parent zeolite, and Au-HBeta(AP) whose BET surface area was the lowest from among all materials. The decrease in surface area observed for the former sample resulted from partial blockade of micropores by small gold nanoparticles. For the latter sample, Au-HBeta(AP), the decrease in surface area was also attributed to blockage effects by small gold particles, however it was accompanied by a decrease in the total pore volume. Presumably, this phenomenon resulted from the presence of silicon species originating from APTMS modifier which additionally blocked the zeolite pores, making N_2_ entry impossible. It is worth noting that the functionalization of HBeta zeolite with APTMS prior to gold deposition resulted not only in a significant decrease in the surface area of micropores, but also in the external surface area of the sample, indicating the location of the modifier on the external surface of the zeolite support.

### 2.3. Gold Particle Size

The size of gold nanoparticles is one of the most important factors determining their activity. Literature data have shown that most often catalytic performance of gold species is much better if Au NPs are smaller [47]. In the present study, the size of gold nanoparticles formed on the surface of all the zeolite samples studied was estimated on the basis of XRD and TEM measurements. The former technique is based on the Scherrer equation which relates the size of crystallites in a solid to the broadening of a peak in a diffraction pattern [48]. This method has one important limitation. Very small gold nanoparticles (with the size much below 5 nm) cannot be identified by XRD. Thus, the materials synthesized in this study were additionally characterized by TEM, which provided information not only about the size, but also the distribution of gold particles.

**Figure 2:**
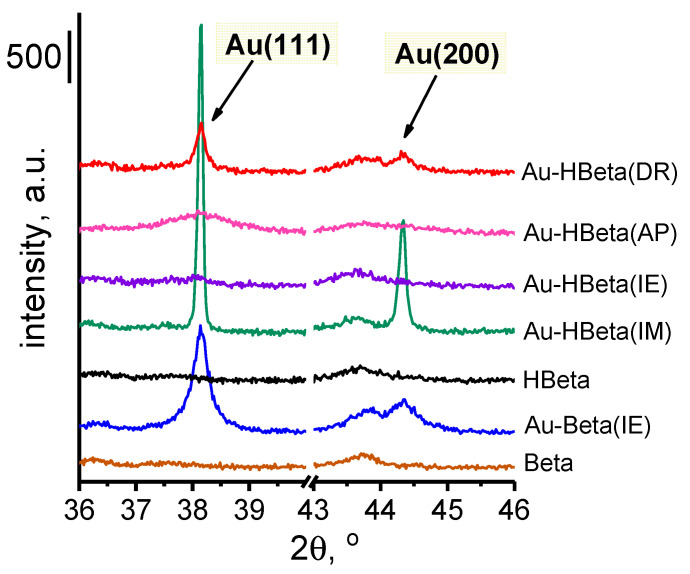
XRD patterns of zeolites presenting diffraction peaks originating from metallic gold.

As can be seen from the XRD data (Figure 2), the most intense and the sharpest diffraction peaks characteristic of metallic gold species were observed for Au-HBeta(IM), indicating that gold nanoparticles loaded on Beta zeolite by wet impregnation were the largest from among those deposited on Beta zeolite by different methods. The average gold particle size estimated for this sample on the basis of XRD data was found to be of 85 nm. On Au-Beta(IE) and Au-HBeta(AP), which contained similar quantities of gold to that found for Au-HBeta(IM), gold nanoparticles were much smaller, and their average size was found to be of 33 and 8 nm, respectively (see Table 1). It is worth noting that the material prepared by the deposition-reduction method (Au-HBeta(DR) exhibited ca. twice as small a gold loading than all above-mentioned samples, but the average size of Au NPs formed on the surface of this material was much larger than that observed for Au-Beta(IE) and Au-HBeta(AP), and was found to be of 52 nm. For Au-HBeta(IE), estimation of gold particle size on the basis of XRD pattern was impossible due to very low intensity of the diffraction peaks typical of gold species. This phenomenon resulted more likely from the very low efficiency of gold introduction by this method (the actual gold loading of 0.2 wt.%; Table 1).

**Figure 3:**
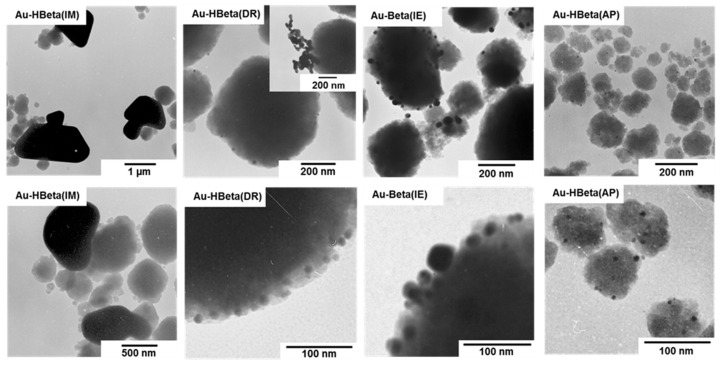
TEM images of prepared materials.

Figure 3 and Supplementary Figures S1 and S2 show the TEM images of gold samples prepared with the use of different gold deposition methods. The largest gold particles were observed for the material prepared by wet impregnation (see Figure 3). Gold particles formed on the surface of this sample were larger that the aggregates of the Beta zeolite. Their size varied in the range from few hundreds nanometers to more than a few micrometers. Much smaller but relatively large gold particles were also identified on the surface of the sample prepared by ion-exchange in the hydrogen form of Beta zeolite.

**Figure 4:**
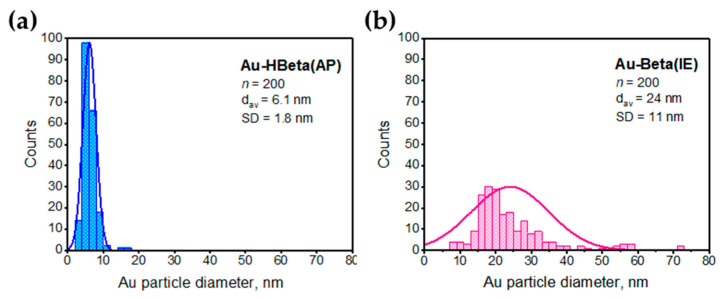
Gold particle size distributions estimated for (**a**) Au-HBeta(AP) and (**b**) Au-Beta(IE) by counting *n* = 200 particle diameters in each sample. The average Au particle diameter (d_av_) and standard deviation (SD) were determined.

Supplementary Figure S1 clearly shows that gold particles in this material formed large aggregates localized only on the surface of selected zeolite particles. Most probably, this phenomenon is a consequence of a very weak interaction between the zeolite support and the gold precursor, which enabled the agglomeration of Au NPs during the calcination step [47,49,50]. It is highly likely that the aggregation of gold species observed for this material resulted from relatively high resistance of the hydrogen form of Beta zeolite to ion exchange, as suggested in Section 2.1. This feature of HBeta support led not only to the low efficiency of gold introduction, but also favored the aggregation of gold species upon high thermal treatment (low stabilization of gold species related to weak interaction with the zeolite matrix). Some agglomeration effect was also observed for the sample prepared by deposition-reduction method. As can be seen in Figure 3 and Supplementary Figure S2, Au-HBeta(DR) contained mainly relatively small gold nanoparticles of the size ranging from 5 to 20 nm, which were homogeneously distributed on the external surface of the zeolite. However, some minor areas where agglomeration of gold species took place were also found on this sample (see Supplementary Figure S2). Interestingly, the formation of large aggregates of gold particles was not observed for the materials prepared by ion-exchange in the ammonium form of Beta zeolite and anchoring of gold species on the support grafted with APTMS. Figure 3 shows that Au NPs formed on the surface of these two samples were relatively small and more homogenously distributed on the zeolite support than gold species in other materials. To get a deeper insight into the influence of gold deposition method on the size of gold nanoparticles we have estimated the gold particle size distribution. Data for Au-HBeta(IM), Au-HBeta(IE) and Au-HBeta(DR) are not included because of a small number of gold particles identified on the TEM images and the formation of large aggregates of Au NPs, which hindered the reliable measurement of gold particle size in the aggregates. The distributions of gold particle size in Au-Beta(IE) and Au-HBeta(AP) are shown in Figure 4. It was found that Au NPs formed on the surface of Au-HBeta(AP) were much smaller and more homogeneous in size than those observed for Au-Beta(IE). The average size of Au NPs in the former sample estimated on the basis of TEM measurements was found to be of 6 nm, which is in a good agreement with the values calculated from XRD patterns. On Au-Beta(IE), the gold particle size distribution was much broader and ranged from ca. 10 nm to ca. 60 nm. The average gold particle size estimated for this material was much larger than that observed for Au-HBeta(AP) catalyst, and was found to be of 24 nm. This value is lower than that calculated on the basis of XRD measurements (24 nm vs. 33 nm, respectively; see Table 1). This observation can be explained by limitations of the XRD technique which allows identification of relatively large gold species only. In other words, if a sample contains both small and large gold nanoparticles, XRD data will allow for estimation of the size of the larger ones.

The results obtained in this study clearly show that the gold deposition method has a significant influence on the size of Au NPs. The largest gold nanoparticles were observed for the sample prepared by wet impregnation, while the smallest Au NPs were formed on the surface of the material prepared by anchoring of gold species on the surface of Beta zeolite grafted with APTMS. Importantly, the gold loading method affected not only the size but also the distribution of gold species. The most homogeneous in size and the most uniform distribution of Au NPs on the zeolite surface was characteristic of Au-HBeta(AP). This shows a great advantage of the organosilane-based method over other ways of gold introduction on Beta zeolite.

### 2.4. Surface and Electronic Properties of Zeolites—XPS Study

To get a deeper insight into oxidation state of gold all the samples were carefully characterized with the use of X-ray photoelectron spectroscopy. As can be seen from the Au 4f region, shown in Supplementary Figure S5, for all materials containing gold, we observed two peaks at binding energy values of ca. 84.2 and 87.9 eV. According to the literature [51], these peaks are assigned to metallic gold species (spin orbitals Au 4f_7/2_ and Au 4f_5/2_, respectively). It is worth noting that there were no significant differences in the positions of these peaks, indicating that, in all the samples, gold species existed in metallic form. The Au 4f peaks observed for the samples containing large gold species (e.g., Au-HBeta(IM)) were expected to be shifted towards lower binding energy values than that found for a material with much smaller gold nanoparticles (e.g., Au-HBeta(AP)). However, such a shift was not observed in this study. Most probably, it is a result of difficulties in the precise determination of the Au 4f peak positions for the materials with very large gold species for which the signal intensity was very low (see Supplementary Figure S5).

**Figure 5:**
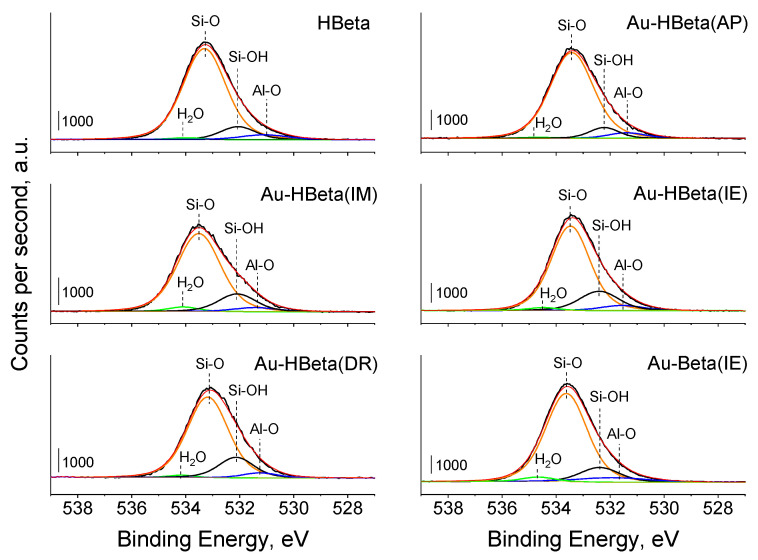
O 1s XP spectra of HBeta and gold-modified zeolites.

In the O 1s region, an intensive peak at ca. 533.5 eV with a tail at a lower binding energies appears (see Figure 5). Deconvolution of this O 1s peak allowed us to distinguish its four components, at ca. 534 eV, 533.5 eV (the dominant), 531.5 eV, and 532.2 eV (detailed positions of individual components and their relative contributions are summarized in Supplementary Table S1). According to the literature [31], the most intensive component at BE of ca. 533.5 eV is characteristic of oxygen species bonded to silicon atom (Si-O). The component at BE of ca. 531.5 eV is assigned to oxygen atoms bonded to aluminum (Al-O), while the peak at BE of 532.2 eV is typical of Si-OH groups. The last component with the lowest intensity at BE of ca. 534 eV, is characteristic of water molecules adsorbed on the zeolite surface. Table S1 clearly shows that gold deposition method has significant impact on the relative contribution of Si-OH species. The lowest contribution of this component was observed for the sample prepared by anchoring of gold species on the surface of Beta zeolite grafted with APTMS. Precise deconvolution of O 1s peak permitted estimation of Si/Al ratio for all the materials. As can be seen from Table 1, Si/Al ratio estimated on the basis of XPS data is in agreement with the values obtained by using ICP. As follows from the results, the loading of gold on the zeolite by deposition-reduction, wet impregnation, or anchoring of gold species on the support grafted with APTMS had negligible influence on Si/Al ratio in these materials. However, for the sample prepared by ion-exchange in the ammonium form of Beta zeolite, a significant decrease in the Si/Al ratio after gold deposition was observed.

### 2.5. Acidity of Zeolites

HBeta zeolite was used as a support for gold introduced with the majority of procedures applied in this work. However, the commercial ammonium form of Beta zeolite was also used for the ion exchange procedure because it allowed deposition of more gold species than HBeta. Therefore, in the characterization of acidity, both zeolite supports, i.e., the commercial ammonium form of Beta and HBeta prepared by calcination, were examined as references for gold containing samples.

**Figure 6:**
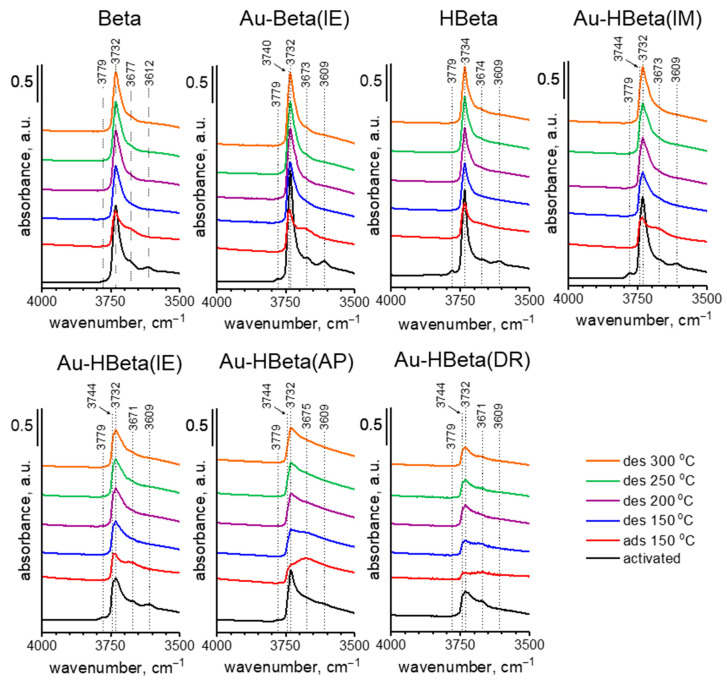
Hydroxyl groups vibration range of FTIR spectra recorded after activation at 350 °C, adsorption (at 150 °C), and desorption of pyridine (at 150, 200, 250, and 300 °C). All the spectra were normalized to the density of a wafer of 10 mg·cm^−2^.

**Figure 7:**
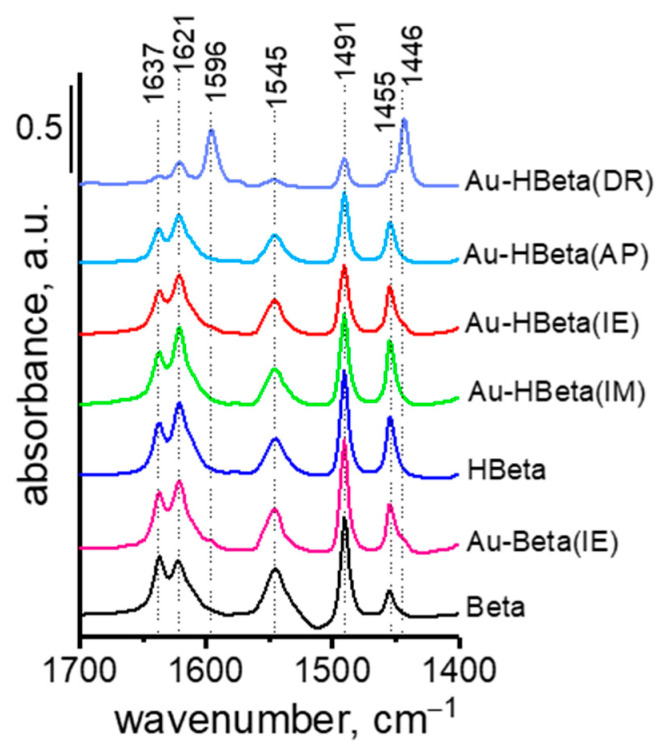
FTIR spectra after desorption of pyridine at 200 °C. All the spectra were obtained by subtraction the spectrum after activation and normalized to the density of a wafer of 10 mg·cm^−2^.

The acidity of the zeolites examined in this study was estimated on the basis of pyridine adsorption at 150 °C followed by evacuation at this temperature and at higher temperatures, interpreted together with FTIR measurements. The infrared spectra of the samples studied are presented in Figure 6; Figure 7 and Supplementary Figure S6. According to the literature, four typical OH vibrations are characteristic of HBeta zeolite. The authors noted the presence of the following OH groups giving rise to the appropriate IR bands: Si-OH at 3746 cm^−1^ (with a shoulder at 3736 cm^−1^), Al-OH at 3781 and 3670 cm^−1^ and a band due to bridging (Al-OH-Si) hydroxyl groups at 3605 cm^−1^. Some authors [52,53,54] have also observed a broad band in the range of 3500–3200 cm^−1^ assigned to hydrogen bonded HO…O species. The spectra presented in Figure 6 show all four bands assigned to activated Beta and HBeta zeolite, but the wavenumbers are shifted in relation to those presented in [55], which can be linked to different Si/Al ratios (~11 in the referred literature vs. 17.8 and 18.4 for Beta and HBeta, respectively, in this work). Thus, the following bands are shown in Figure 6 for HBeta (and Beta) zeolite: ~3610 cm^−1^ (3614 cm^−1^), typical of Brønsted acid sites, 3779 cm^−1^ and 3674 cm^−1^ (3678 cm^−1^) interpreted by some authors [53,54] as coming from acidic hydroxyls, but by others [55] assigned to two kinds of non-acidic Al-OH groups (typical of extra-framework aluminum species), and 3734 cm^−1^ (3732 cm^−1^), a band characteristic of silanol groups. The position of the latter band suggests that it originates from hydrogen bonded silanol groups (due to their high concentration). It should be noted that the band at 3779 cm^−1^ in the spectrum of Beta zeolite was of negligible intensity in comparison with that in the spectrum of HBeta. This means that the applied procedure of preparation of the hydrogen form of Beta zeolite (HBeta) resulted in an increase in the amount of Al-OH species characterized by vibrations giving the IR band at this wavenumber. The key reason for this could be a high temperature of calcination (550 °C) in the procedure of HBeta formation, leading to a higher degree of dealumination, and thus to an increase in Al-OH species.

Pyridine adsorption at 150 °C, followed by evacuation at the same temperature, changed the spectrum in the hydroxyl region (Figure 6) and caused the appearance of bands in the region characteristic of stretching vibrations in pyridine molecules (1700 cm^−1^–1400 cm^−1^; Figure 7 and Supplementary Figure S6). A decrease in the intensity of the band assigned to silanol groups (3734 cm^−1^) was accompanied by the appearance of two bands characteristic of hydrogen bonded pyridine (1446 cm^−1^ and 1596 cm^−1^ typical of symmetric and antisymmetric vibrational bands, respectively [55,56,57,58]). With an increasing temperature of evacuation, the band from silanol groups was rebuilt to a high degree and those coming from vibrations in hydrogen bonded pyridine (1446 cm^−1^ and 1596 cm^−1^) disappeared after evacuation at 200 °C, which implies that the silanol groups take part in a weak chemisorption of pyridine. The other three bands characteristic of hydroxyls, at 3780 cm^−1^, 3673 cm^−1^ (Al-OH groups), and 3609 cm^−1^ (typical of BAS, Al-OH-Si), disappeared after pyridine adsorption at 150 °C. At the same time, the following pairs of bands were recorded: 1455 cm^−1^ and 1621 cm^−1^ originating from symmetric and antisymmetric stretching vibrations in pyridine coordinatively bonded to LAS as well as at 1545 cm^−1^ and 1637 cm^−1^ characteristic of vibrations in pyridine cations formed by the protonation of pyridine on BAS. The most intensive band at 1491 cm^−1^ comes from vibrations in pyridine chemisorbed on both LAS and BAS. Both types of chemisorbed pyridine (on LAS and BAS) were strongly bound to the surface of HBeta as evidenced from still intensive bands characteristic of chemisorbed species after evacuation at 300 °C. The character of the spectrum of Beta zeolite in the range of vibrational bands of adsorbed pyridine is the same as that of HBeta zeolite. The only difference is the relationship between the number of LAS and BAS occupied by pyridine. Their number was estimated from the intensity of infrared bands characteristic of symmetric vibration in pyridine protonated on BAS (1545 cm^−1^) and coordinated to LAS (1455 cm^−1^) using the extinction coefficients of 0.044 cm^2^·μmol^−1^ for BAS and 0.165 cm^2^·μmol^−1^ for LAS [59]. The results are presented in Table 3. For the band at 1455 cm^−1^, correct calculation of the number of LAS occupied by chemisorbed pyridine was possible for the Au-Beta(IE) sample only from evacuation at 250 °C because the zeolite evacuated at this temperature totally lost the band coming from hydrogen bonded pyridine (1445 cm^−1^) which partially overlapped the band at 1455 cm^−1^. Therefore, in Table 3, the amount of LAS occupied by chemisorbed pyridine is shown only for zeolites evacuated at 250 °C after pyridine adsorption.

The ratio of the amount of pyridine strongly chemisorbed on BAS in HBeta after evacuation at 300 °C to that chemisorbed after evacuation at 250 °C was 0.83. This value is very similar to the literature [60] value of 0.85 for HBeta at a similar ratio Si/Al = 20. It indicates that our results are compatible with literature data although the real amounts of chemisorbed pyridine are different. However, the numbers of BAS and LAS reflected by moles of chemisorbed pyridine depend mainly on the activation temperature (350 °C in our study and 450 °C in [60]). The higher the activation temperature, the lower the number of BAS and the higher the content of LAS.

The influence of the method of gold loading on the number and strength of LAS and BAS in the zeolite matrices can be estimated on the basis of the data shown in Table 3 and the spectra presented in Figure 6 and Figure 7 and Supplementary Figure S6. The character of the spectra of HBeta zeolites modified with gold in the hydroxyl region is considerably different than that of the spectrum of the pristine support. The exception to this is Au-HBeta(IM), whose spectrum is similar to that of activated HBeta although the band at 3732 cm^−1^ is wider from the side of higher wavenumbers for the gold containing zeolite. For gold modified Beta zeolite (Au-Beta(IE)) the spectrum of activated materials is the same for gold containing sample and the pure commercial Beta zeolite. Significant and similar changes in the spectra of activated Au-HBeta(DR) and Au-HBeta(IE) in comparison to the spectrum of activated HBeta are well visible in Figure 6. A new infrared band at 3744 cm^−1^ is clearly indicated. The position of this band suggests that it originates from silanol groups but located at different sites than that to which the band at 3732 cm^−1^ in the spectrum of activated HBeta is assigned. It is strongly supposed that this band comes from isolated silanol groups. The character of the spectra allowed us to conclude that the treatment of the zeolite studied in both modification methods led to generation of different types of Si-OH groups, i.e., surface isolated silanols. For Au-HBeta(DR) the reason for the formation of such silanols can be the treatment with reducing agent (sodium borohydride) which releases hydrogens interacting with the zeolite structure. The synthesis of Au-HBeta(IE) required 18 h treatment with chloroauric acid, which led to the interaction of acidic protons (released from Si-OH-Al BAS during ion exchange) with the zeolite surface. Interestingly, such changes did not occur when the ammonium form of the zeolite (commercial Beta) was subjected to the same procedure. The conclusion about new silanol groups formed during the HBeta zeolite modification with Au species is supported by XPS spectra in the O 1s region showing increasing intensity of the peak characteristic of Si-OH (Figure 5, Supplementary Table S1). The character of the XPS spectrum of Au-HBeta(AP) is different than that described for Au-HBeta(DR) and Au-HBeta(IE). Functionalization of the zeolite with APTMS prior to anchoring of chloroauric acid reduced the amounts of all types of hydroxyls observed for HBeta. This observation can be reasonably explained by assuming that all types of surface hydroxyls took part in anchoring of APTMS. Hydroxyls were not fully rebuilt after calcination of gold/APTMS-containing zeolite. For Au-HBeta(AP), the new type of silanols did not give a new signal in the spectrum of activated sample, but a shoulder at ca. 3744 cm^−1^ was slightly noticeable after pyridine adsorption. A similar shoulder also appeared in the spectra of Au-HBeta(IM) after pyridine adsorption and desorption at different temperatures. It should be emphasized that the surface silanol groups giving the band at 3732 cm^−1^ easier interacted with pyridine (a higher decrease in intensity of this band after pyridine adsorption) than the newly generated Si-OH groups (3744 cm^−1^) observed for the samples Au-HBeta(DR) and Au-HBeta(IE), whose presence was manifested as a shoulder for Au-HBeta(AP) and Au-HBeta(IM) (Figure 6). Interaction of these species with pyridine resulted in reducing the intensity of the band at 3732 cm^−1^, which allowed observation of the band at ca. 3744 cm^−1^ coming from isolated silanols. Thus one can conclude that modification of HBeta with gold species is accompanied by changes in the zeolite surface properties (formation of new isolated silanol groups) and the level of these changes depends on the preparation procedure, it is higher for the method based on ion exchange and for deposition-reduction method. Modification of the ammonium form of Beta zeolite by ion exchange with chloroauric solution did not lead to generation of new isolated silanols.

For Au-HBeta(DR) additional changes in the spectra in the hydroxyl region were observed. The band at 3610 cm^−1^ characteristic of bridged Al-OH-Si BAS typical of HBeta zeolite disappeared after the sample modification with gold species. This disappearance must have been a result of the zeolite treatment with sodium borohydride (the reducing agent) which led to the exchange of protons from the bridged Al-OH-Si by sodium cations and in this way reduced significantly the number of BAS. This process resulted in a drastic decrease in the number of BAS occupied by adsorbed pyridine (Table 3). Thus, the number of BAS given in Table 3 for this sample is determined by the hydroxyl groups responsible for the band at 3670 cm^−1^ which was assigned to acidic species by the authors of [48,49]. The percentage of pyridine desorbed at 300 °C in relation to the amount of pyridine chemisorbed after evacuation at 200 °C for Au-HBeta(DR) is much lower than for all other samples presented in Table 3. This percentage gives an estimate of the strength of BAS. The higher the percent of desorbed pyridine, the lower the strength of BAS. Thus, BAS characterized by the infrared band at 3670 cm^−1^ is stronger than the bridged hydroxyls characterized by the band at 3610 cm^−1^. The plots shown in Supplementary Figure S7 are drawn taking into account the amount of pyridine chemisorbed after evacuation at 200 °C. The amount of pyridine chemisorbed on BAS increased after evacuation at 200 °C when pyridine hydrogen bonded to silanol groups disappeared (see the bands at 1446 cm^−1^ and 1596 cm^−1^; Supplementary Figures S6 and S7 and Table 3). This phenomenon implies that hydrogen bonded pyridine hindered the access to a part of BAS. When the hydrogen bonded pyridine was desorbed at 200 °C, some of the previously unavailable BAS chemisorbed pyridine during diffusion of this base within the pores. For all materials, the number of BAS occupied by pyridine was the highest after evacuation at 200 °C. Therefore, these numbers were used as 100% of pyridine chemisorbed for estimation of the strength of acidity on the basis of pyridine adsorption drop after evacuation at 300 °C.

Figure 7 and Supplementary Figure S6 shows that pyridine adsorption on all gold-containing zeolites resulted in the appearance of the same bands in the infrared spectra as pyridine adsorbed on the supports, Beta and HBeta. The difference was in the intensities of these bands, which reflects the number of acidic sites involved in pyridine adsorption. The number of BAS can be estimated on the basis of pyridine chemisorbed after evacuation at 200 °C (Table 3). After evacuation at this temperature the maximum of pyridine chemisorbed on BAS was recorded as shown in Supplementary Figure S7. It is clear that impregnation of HBeta with chloroauric acid (Au-HBeta(IM) sample) did not change the number of BAS but it increased their strength (the difference in the amount of pyridine desorbed from BAS after evacuation at 200 °C and 300 °C was 34% for HBeta and 24% for Au-HBeta(IM)). For all other zeolites, the number of BAS decreased after modification with gold species. Interestingly, a significant decrease in the BAS number (by ca. ¼) and strength was observed for Au-HBeta(AP), which can result from the functionalization of the zeolite with basic APTMS.

As concerns Lewis acid sites, their number increased ca. twice for Au-Beta(IE) and by 14% for Au-HBeta(IM) in comparison with the number of LAS in pristine supports, Beta and HBeta, respectively. For the latter sample the increase in the number of LAS cannot be due to the dehydroxylation because the number of BAS was the same for both HBeta and Au-HBeta(IM). The increase in the amount of pyridine chemisorbed on LAS can be attributed to the interaction of pyridine with gold NPs which are electron acceptors and act as Lewis acids as evidenced in [12,61]. Interestingly, for other gold containing materials, especially for Au-HBeta(AP) and Au-HBeta(DR), a decrease in the number of LAS occupied by pyridine was noted (Table 3). This decrease was accompanied by a decrease in the number of BAS. This observation can be interpreted as in the paper of Behraves et al. [12], who claimed that it can result either from the interaction of very small metal particles with LAS in the support or the leaching of Al from the support surface during the gold deposition procedure. In this study the presence of very small Au NPs (~2 nm; Figure 4a) was found for Au-HBeta(AP) whereas in Au-HBeta(DR) the surface Si/Al ratio (19.4; measured from XPS; Table 1) was higher in comparison to that of HBeta (17.3) indicating leaching of surface Al species. The decrease in BAS number can originate from different causes depending on the modification method. For Au-HBeta(AP) the interaction between the amine groups from APTMS with BAS towards the formation of protonated amine groups decreased the BAS number, whereas in the deposition-reduction procedure (Au-HBeta(DR)) sodium cations from sodium borohydride (reducing agent) could exchange protons from BAS.

Finally, the BAS/LAS ratio should be considered as it is often used as an indicator of acidity of bifunctional materials. The highest BAS/LAS ratio was found for gold zeolites modified by the ion exchange procedure (2.90 and 2.46 for Au-Beta(IE) and Au-HBeta(IE), respectively), whereas the lowest one for Au-HBeta(DR).

The above analysis of acidic properties of gold-containing zeolites clearly shows the importance of proper choice of gold modification procedure for the preparation of the bifunctional materials (redox–acidic) addressed to desired applications. The preliminary study indicated the effectiveness of these materials in base-free glucose oxidation. As it was pointed out in [62], catalysts containing relatively large/medium sized gold particles can be promising candidates for this reaction.

## 3. Materials and Methods

All materials used in this work were prepared from the commercial ammonium form zeolite Beta (Alfa Aesar, Lancashire, UK, Si/Al = 19). The hydrogen form of Beta (HBeta) was obtained by calcination of the pristine ammonium form of Beta zeolite at 550 °C for 15 h (heating rate: 1 °C·min^−1^). The as-prepared zeolite supports were further modified by loading gold by using four different methods: (i) wet impregnation, (ii) ion exchange, (iii) deposition-reduction, and (iv) anchoring of gold species on the surface of zeolite support grafted with organosilane ((3-aminopropyl)trimethoxysilane). In all the materials, chloroauric acid (HAuCl_4_·*x*H_2_O) was used as a gold source and the assumed gold loading was 2 wt.%. The synthesis of all the materials is described below.

### 3.1. Preparation of Au-HBeta(IE), Au-Beta(IE)_18h and Au-Beta(IE) by Ion Exchange Method

The procedure was founded on the Sobczak et al. work [63]. A portion of 125 mL of gold precursor (HAuCl_4_, Sigma Aldrich, St. Louis, MO, USA, 99.995%) solution of ca. 8.2 × 10^−4^ mol·L^−1^ was prepared. Then pH was adjusted with the use of diluted NaOH solution to 6.0 ± 0.1. Next, 1 g of HBeta was added to the as-prepared solution. The mixture was then heated for 18 h at 80 °C under reflux (mixing rate: 600 rpm). After the heating step, the solid was separated by centrifugation and washed with 40 mL of deionized water. Finally the sample was dried for 12 h at 80 °C and calcined for 2 h at 400 °C.

Au-Beta(IE)_18h and Au-Beta(IE) samples were prepared by the same procedure as above, but with the use of ammonium form of Beta instead of HBeta. Furthermore, Au-Beta(IE) was obtained on a larger scale. For this purpose, 12 g of Beta ammonium form was added to 1 L of gold precursor solution (pH of the solution was adjusted to 6.0 ± 0.1 by using NaOH prior to zeolite addition). In this preparation protocol, the heating step was extended from 18 to 42 h.

### 3.2. Preparation of Au-HBeta(IM) by Wet Impregnation Method

The wet impregnation method of Au introduction was inspired by the work [64]. Before gold introduction, HBeta was previously dried overnight at 200 °C in order to remove adsorbed water and to unblock the pores. Next, a required amount of gold precursor was dissolved in 15 mL of deionized water. The previously dried zeolite was then added to the HAuCl_4_ solution and the mixture was stirred. Following 5 min of stirring at room temperature and 5 min of sonication, the mixture was transferred to a rotary evaporator and water was removed. The as-obtained yellow solid was further dried overnight at 80 °C and calcined for 3 h at 500 °C.

### 3.3. Preparation of Au-HBeta(DR) by Deposition-Reduction Method

The procedure of deposition-reduction was similar to that described in [19] with some modification. HBeta zeolite was dispersed in deionized water (1 g per 20 mL). The pH of as-prepared mixture was adjusted to 4.0 ± 0.2 with the use of diluted nitric acid. Then, a required volume of gold precursor solution (3.6 × 10^−2^ mol·L^−1^) was added. The yellowish suspension was stirred for 1 h at room temperature (600 rpm). Meanwhile, the aqueous solution of sodium borohydride (NaBH_4_, Sigma Aldrich, >98%) was prepared (the molar ratio of reducing agent to gold precursor was fixed to be 10:1). After the addition of NaBH_4_ solution into zeolite suspension, the mixture turned purple. Following 30 min of stirring at room temperature the solid was separated by centrifugation and then washed 6 times with deionized water. Finally, the as-prepared material was dried overnight (80 °C) and calcined for 2 h at 400 °C.

### 3.4. Preparation of Au-HBeta(AP) by Anchoring of Gold Species on APTMS-Grafted HBeta

The procedure is based on that proposed by Wolski et al. [15]. In the first step of the synthesis, HBeta zeolite was grafted with APTMS (Aldrich). For this purpose the zeolite support was refluxed in toluene solution of APTMS (200 mL of toluene, 2.5 g of organosilane per 1 g of HBeta) for 18 h at ca. 100 °C. Then, the as-prepared material was recovered by filtration, washed in dry toluene (200 mL), water (100 mL) and acetonitrile (20 mL) and finally dried overnight at 80 °C. The dried organosilane-modified zeolite served as a support for anchoring gold species. The support was dispersed in deionized water (1.5 g per 25 mL of water) and then HAuCl_4_ aqua solution was added (assumed gold loading: 2 wt.% in relation to mass of the dry support without the modifier). The as-prepared mixture was stirred for 1 h at room temperature. Next, the solid was separated by filtration and washed with water. In the next step, the as-prepared solid sample was redispersed and reduced using aqueous solution of sodium borohydride. The molar ratio of reducing agent to gold precursor was fixed to be 10:1. Following 20 min of stirring at room temperature, the gold-containing material was separated by filtration followed by washing with deionized water until the negative silver nitrate test for borohydride. Finally, the as-prepared material was dried overnight (80 °C) and calcined for 4 h at 500 °C.

### 3.5. Characterization Techniques

The content of metals in the catalysts was determined by Inductively Coupled Plasma Optical Emission Spectrometry (ICP-OES 9820 Shimadzu, Kyoto, Japan). In order to determine the actual gold loading, the samples (approximately 100 mg) were mineralized in the mixture of acids consisting of concentrated HNO_3_ and HF (all acids supplied by Merck, Darmstadt, Germany). Digestion was carried out using a Microwave Reaction System (Multiwave PRO equipped with the acid digestion rotor 8NXF100). For the analysis of Si and Al, all the samples (approximately 100 mg) were weighted and mineralized using a mixture of acids composed of 6 mL of HCl, 2 mL of HNO_3_ and 1 mL of HF (all acids supplied by Sigma-Aldrich, St. Louis, MO, USA). Digestion was carried out using a Microwave Reaction System (Anton Paar, Graz, Austria). The XRD patterns were recorded on a D8 Advance diffractometer (Bruker, Karlsruhe, Germany) using CuKα radiation (λ = 0.154 nm), with a step size of 0.02° in the 2θ range of 6–60°. The N_2_ adsorption-desorption isotherms were obtained at −196 °C using a Micromeritics ASAP 2020 Physisorption Analyzer. Before measurements, the samples were degassed at 200 °C for 4 h. The surface area of the materials obtained was calculated by the BET method.

Diffuse reflectance UV–vis spectra (DR UV–vis) were recorded on a Varian Cary 300 Scan spectrophotometer equipped with a diffuse reflectance accessory. The spectra were recorded at room temperature in the range from 200 to 800 nm. Spectralon was used as the reference material. X-ray photoelectron spectroscopy (XPS) was performed using an ultra-high vacuum photoelectron spectrometer based on Phoibos150 NAP analyzer (Specs, Berlin, Germany). The analysis chamber was operated under vacuum with a pressure close to 5 × 10^−9^ mbar and the sample was irradiated with a monochromatic Al Kα (1486.6 eV) radiation. Any charging that occurred during the measurements (due to incomplete neutralization of ejected surface electrons) was compensated by rigidly shifting the entire spectrum by a distance needed to set the binding energy of the C 1s assigned to adventitious carbon to the assumed value of 284.8 eV. For transmission electron microscopy (TEM) measurements the powders were deposited on a grid covered with a holey carbon film and transferred to a JEOL 2000 electron microscope operating at 80 kV. The gold particle size distribution was calculated using the Image J software.

Pyridine (Py) adsorption measurements were performed on powdered samples pressed under low pressure into thin wafers of ca. 10–20 mg·cm^−2^ and placed inside a quartz cell equipped with NaCl windows. Before measurements, the catalysts were evacuated at 350 °C for 2 h in order to remove water (activation step). Then, Py was introduced into the cell at 150 °C and after the saturation, the excess of pyridine was removed by degassing in vacuum at 150 °C for 5 min (adsorption step). The next degassing was performed at 150, 200, 250, and 300 °C in vacuum for 30 min at each temperature (evacuation steps). Infrared spectra were recorded with a Bruker Inveno S spectrometer at room temperature in the range from 4000 to 400 cm^−1^ after activation, adsorption of Py, and after 30 min of evacuation at each above-mentioned temperature.

## 4. Conclusions

HBeta zeolite was the parent support for most modifications performed in this study. As the efficiency of gold deposition on this zeolite by the ion exchange procedure was very low (0.2 wt.%), the commercial ammonium form of Beta zeolite was applied for application of this technique. Thus, Au-HBeta(IE) zeolite will not be considered in further conclusions because of a very low gold loading. The efficiency of gold deposition by all other methods was much higher and almost the same for Au-Beta(IE), Au-HBeta(IM), Au-HBeta(AP), whereas for Au-HBeta(DR) it was ca. twice lower.

The main focus of this work was on changes in the zeolite properties caused by modification with gold species by different procedures. It has been found that some changes in zeolite properties were caused directly by gold modification procedure and some of them resulted from the size of Au NPs, which was determined by the technique of gold deposition. As concerns the zeolite composition, the impregnation method did not influence it because it involves a simple and short operation. This is not the case of the ion exchange procedure, which required a very long (42 h) treatment of Beta zeolite with the solution of chloroauric acid with pH adjusted by NaOH, which resulted in partial desilication of the zeolite leading to a significant decrease in Si/Al ratio. Such drop in Si/Al ratio resulted in changing the acidic properties of Au-Beta(IE) leading to its highest BAS/LAS ratio from among all samples studied. The other modification procedures did not change considerably the Si/Al ratio. The textural parameters like surface area and pore volume strongly depended on Au particle sizes, affected by the gold deposition method. The largest gold NPs were formed when the wet impregnation method was used for Au deposition (Au-HBeta(IM)—average size 88 nm from XRD). Relatively large Au NPs were also observed in the sample prepared by the ion exchange procedure (Au-Beta(IE)—32 nm from XRD and 24 nm from TEM). The smallest gold particles appeared on the sample obtained in the two-step modification, first functionalization with APTMS and next gold anchoring (Au-HBeta(AP)—8 nm from XRD and 6 nm from TEM). The most homogeneous Au NPs distribution was observed for the latter material. The deposition-reduction method (Au-HBeta(DR) led to a wide particle size distribution with the domination of the fraction of 5–20 nm. Small gold particles caused a blockade of micropores in the zeolite structure and decreased the surface area and pore volume. The highest decrease in these textural parameters was noted for Au-HBeta(AP), but it was also observed to a lesser extent for Au-HBeta(DR), as follows from the sizes of gold particles.

The size of gold particles also determined the number of Lewis acid sites in the modified zeolite. The highest increase in the number of LAS in relation to the parent zeolite occurred in the samples containing the largest gold particles, i.e., in Au-HBeta(IM) and Au-Beta(IE). Strongly electronegative gold species in large crystals acted as electron acceptors, i.e., as Lewis acid sites which were added to the number of LAS coming from the zeolite surface. On the other hand, very small gold particles interact with LAS in pores, lowering the number of LAS accessible for bases. This effect was most visible for Au-HBeta(AP). As concerns Brønsted acid sites, their number and strength are determined directly by the procedure applied for gold deposition. The methods in which zeolite treatment with additional substance was used (sodium borohydride in the deposition-reduction technique and APTMS in the gold loading on functionalized zeolite) led to a significant decrease in the BAS number because of the interaction of these additional compounds with acidic hydroxyls. This effect was the highest for the samples obtained by the deposition-reduction method. The strength of BAS significantly increased when the deposition-reduction method was used, which resulted from the reduction of BAS number as a consequence of interaction with sodium cations. This reduction enhanced the strength of the remaining acidic hydroxyls.

The results presented in this paper are meant to help choosing the optimum gold deposition method to obtain materials with the desired bifunctional redox-acid properties and reveal the changes in zeolite properties resulting from application of a specific method.

## Figures and Tables

**Table 1:** Characteristics of materials used in this study.

Sample	Au Content ^a^ [wt.% Au]	Si/Al Molar Ratio (ICP-OES)	Si/Al Molar Ratio ^b^ (XPS)	*d*_Au_ (XRD) ^c^ [nm]	*d*_Au_ (TEM) [nm]
Beta	-	17.8	-	-	-
Au-Beta(IE)	1.5	12.7	11.7	32	24
Au-Beta(IE)_18h	1.2	16.9	-	-	-
HBeta	-	18.4	17.3	-	-
Au-HBeta(IM)	1.5	18.5	18.2	88	- ^d^
Au-HBeta(IE)	0.2	17.5	14.7	- ^e^	- ^f^
Au-HBeta(AP)	1.4	18.6	17.9	8	6
Au-HBeta(DR)	0.7	18.4	19.4	52	- ^f^

^a^ estimated from ICP. ^b^ estimated from XPS data on the basis of deconvoluted O 1s peak [31]. ^c^ estimated from (111) diffraction peak using Scherrer equation. ^d^ insufficient data to perform statistical analysis because of very large size of Au NPs. ^e^ peak at the noise level; impossible to evaluate Au NPs size. ^f^ average gold particle size not estimated because of presence of smaller particles in the range 5–20 nm together with large gold aggregates where precise measurement of particle size was not possible. For more details please see Supplementary Data (Figures S1 and S2).

**Table 2:** Textural properties of investigated materials.

Sample	BET Surface Area [m^2^·g^−1^]	t-Plot Micropore Area [m^2^·g^−1^]	t-Plot External Surface Area [m^2^·g^−1^]	BJH Culminative Volume of Pores (Adsorption Branch) [cm^3^·g^−1^]
Beta	521	379	141	0.14
Au-Beta(IE)	516	349	167	0.16
HBeta	527	377	149	0.13
Au-HBeta(IM)	492	342	150	0.14
Au-HBeta(IE)	520	344	176	0.16
Au-HBeta(AP)	360	260	99	0.11
Au-HBeta(DR)	466	328	138	0.13

**Table 3:** Content of Brønsted (BAS) and Lewis (LAS) acid sites occupied by pyridine after desorption at different temperatures. The calculation based on intensity of IR bands (1545 cm^−1^ for BAS and 1455 cm^−1^ for LAS) and extinction coefficients of 0.044 cm^2^ μmol^−1^ for BAS and 0.165 cm^2^ μmol^−1^ for LAS from [59].

Sample	Evacuation Temp. [°C]	No. of BAS Occupied by Pyridine [μmol·g^−1^]	Pyridine Desorbed at 300 °C from BAS [%] ^a^	No. of LAS Occupied by Pyridine after Evacuation at 250 °C [μmol·g^−1^]	BAS/LAS Ratio after Evacuation at 250 °C
Beta	150	594	26	87	7.03
	200	694			
	250	612			
	300	515			
Au-Beta(IE)	150	475	26	166	2.90
	200	522			
	250	481			
	300	389			
HBeta	150	434	34	196	1.84
	200	452			
	250	360			
	300	297			
Au-HBeta(IM)	150	431	24	223	1.80
	200	455			
	250	401			
	300	345			
Au-HBeta(IE)	150	383	27	160	2.46
	200	429			
	250	394			
	300	313			
Au-HBeta(AP)	150	339	38	137	2.01
	200	345			
	250	275			
	300	215			
Au-HBeta(DR)	150	64	12	119	0.76
	200	87			
	250	91			
	300	77			

^a^ Related to the amount of pyridine chemisorbed after evacuation at 200 °C.

## Supplementary Materials

The following are available online, Figure S1: TEM images of Au-HBeta(IE), Figure S2: TEM images of Au-HBeta(DR), Figure S3: UV-vis spectra of materials, Figure S4: The low-temperature N_2_ adsorption-desorption isotherms for examined materials, Figure S5: Au 4f XP spectra of HBeta and gold-modified zeolites, Table S1: Relative contribution of individual oxygen species estimated from deconvoluted O 1s XP spectra, Figure S6: Pyridine ring vibration range of FTIR spectra recorded after adsorption (at 150 °C), and desorption of pyridine (at 150, 200, 250, and 300 °C), Figure S7: Variations of the amount of Py adsorbed on BAS in relation to evacuation temperature (150, 200, 250, and 300 °C).
