# Supplementary material for: Lights and Shadows of Gold Introduction into Beta Zeolite
Source: Molecules. 2020 Dec 8;25(24):5781. doi: 10.3390/molecules25245781 (PMC7762595; doi:10.3390/molecules25245781)
Supplement: Supplementary file 1 [file molecules-25-05781-s001.pdf]

Supplementary Materials

for Molecules

Lights and shadows of gold introduction into Beta zeolite

Adrian Walkowiak*, Lukasz Wolski, Maria Ziolek

Adam Mickiewicz University, Poznań, Faculty of Chemistry, Uniwersytetu Poznańskiego 8, 61-614 Poznań, Poland

** corresponding author*

e-mail: adrian.walkowiak@amu.edu.pl

List of content:

Figure S1. TEM images of Au-HBeta(IE). The red circles indicate the regions where accumulation of gold species resulted in formation of large gold aggregates. S1

Figure S2. TEM images of Au-HBeta(DR). The inset in **(A)** presents distribution of gold particles which were not aggregated, while the red circle in **(B)** indicates the region where gold particles were fused into large aggregates consisted of Au NPs of different sizes. S2

Figure S3. UV-vis spectra of materials. S3

Figure S4. The low-temperature N_2 adsorption-desorption isotherms for examined materials. S4

Figure S5. Au 4f XP spectra of HBeta and gold-modified zeolites. S5

Table S1. Relative contribution of individual oxygen species estimated from deconvoluted O1s XP spectra [3]... S6

Figure S6. Pyridine ring vibration range of FTIR spectra recorded after adsorption (at 150 °C), and desorption of pyridine (at 150, 200, 250, and 300 °C). All the spectra were obtained by subtraction the spectrum after activation and normalized to the density of a wafer of 10 mg·cm^{-2}. S7

Figure S7. Variations of the amount of pyridine (Py) adsorbed on BAS in relation to evacuation temperature (150, 200, 250, and 300 °C). The calculation based on intensity of 1545 cm^{-1} IR band and extinction coefficient of 0.044 cm^2 μmol^{-1} (from [4]). S8

References: S8

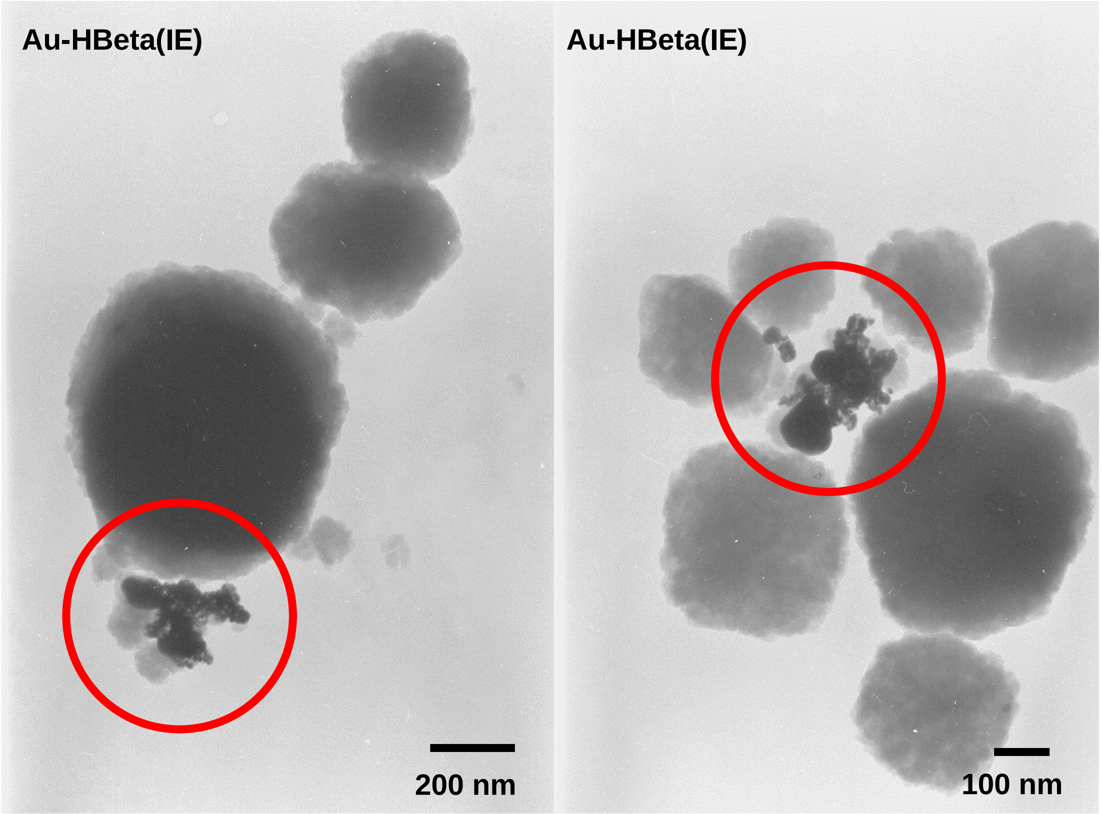

Figure S1. TEM images of Au-HBeta(IE). The red circles indicate the regions where accumulation of gold species resulted in formation of large gold aggregates.

As can be seen from Figure. S1, gold particles in Au-HBeta(IE) were not distributed homogeneously on the catalyst surface and they formed large aggregates consisted of many particles of different sizes.

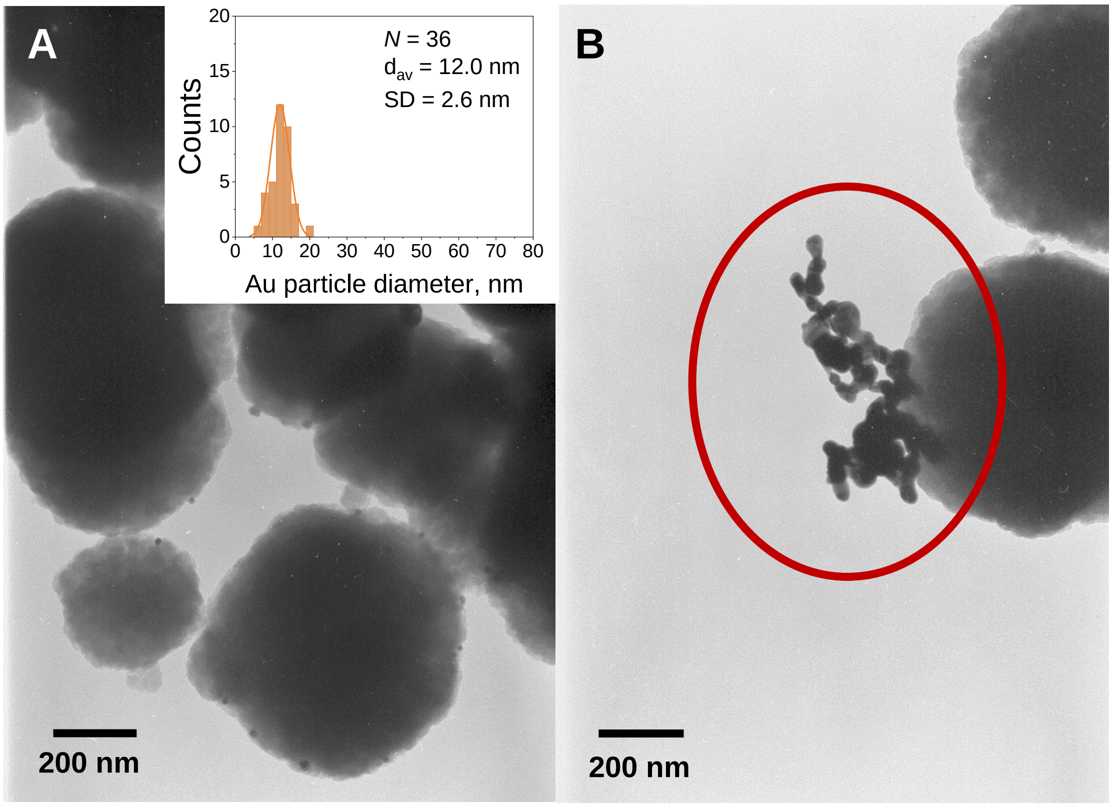

Figure S2. TEM images of Au-HBeta(DR). The inset in **(A)** presents distribution of gold particles which were not aggregated, while the red circle in **(B)** indicates the region where gold particles were fused into large aggregates consisted of Au NPs of different sizes.

TEM images of Au-HBeta(DR) revealed that this sample contained both relatively small gold nanoparticles in the range from ca. 5 to 20 nm (average size: 12 nm; see Figure S2 A) and large gold aggregates shown in Figure S2 B (the latter were sparse). The aggregates consisted of gold particles of different sizes fused into irregular structure where reliable estimation of particle size was impossible. For this reason, we have not estimated the gold particle size distribution for this material.

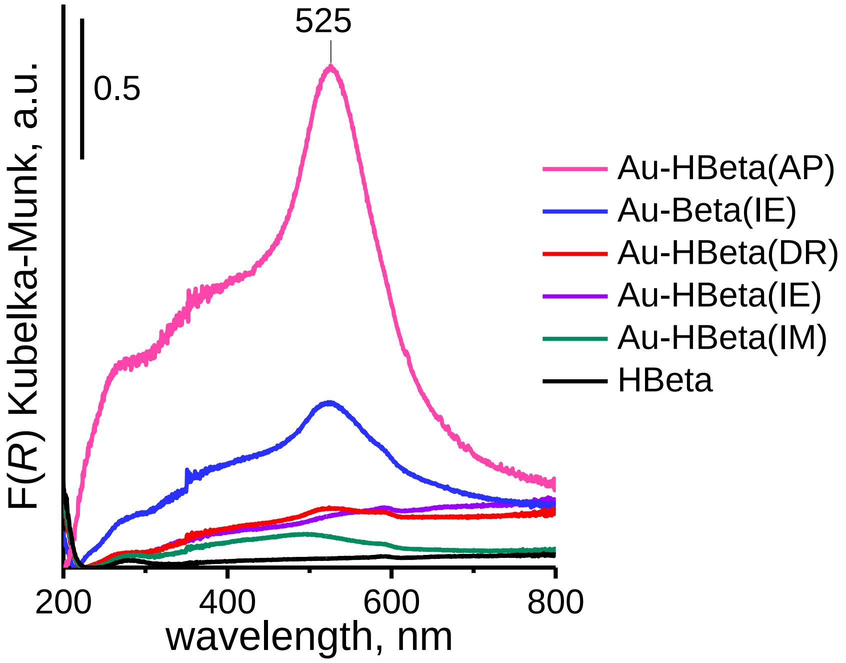

Figure S3. UV-vis spectra of materials.

As can be seen from Figure S3, deposition of gold on Beta zeolite led to appearance of a new absorbance band with maximum intensity at ca. 525 nm. According to literature [1,2] this band is characteristic of surface plasmon resonance of metallic gold nanoparticles and confirms successful loading of gold species on the surface of all the samples.

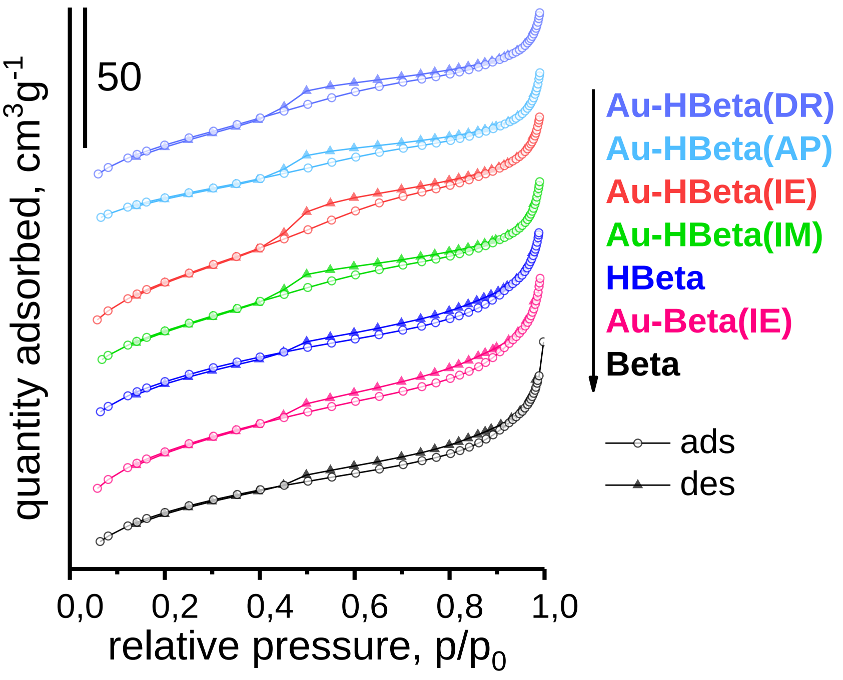

Figure S4. The low-temperature N_2 adsorption-desorption isotherms for examined materials.

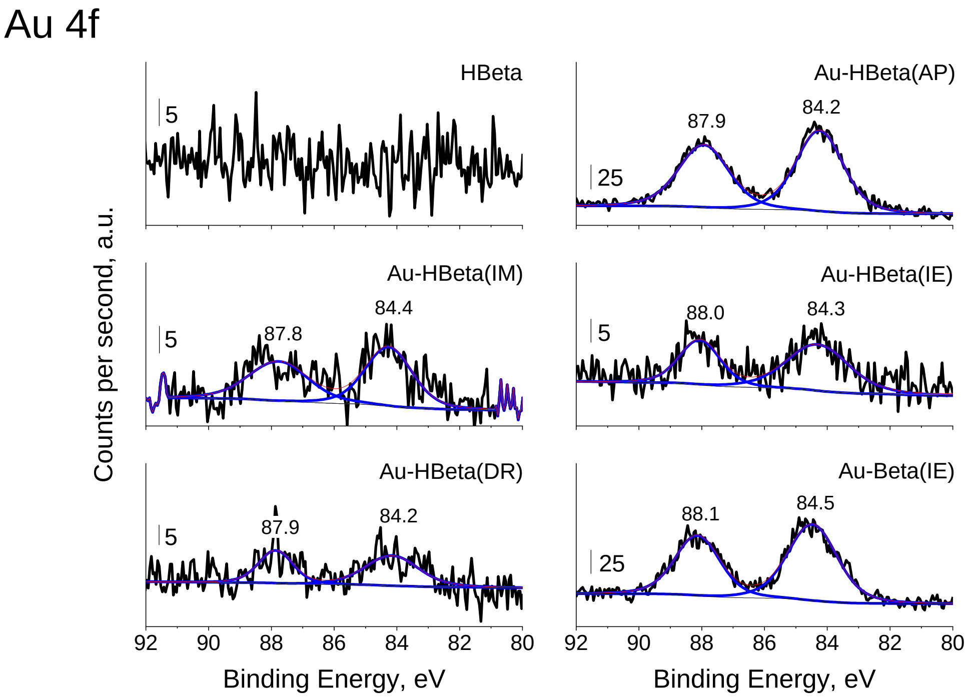

Figure S5. Au 4f XP spectra of HBeta and gold-modified zeolites.

Table S1. Relative contribution of individual oxygen species estimated from deconvoluted O1s XP spectra [3].

Sample		Si–O	Al–O	Si–OH	H_2O
HBeta	BE [eV]	533.3	531.1	532.1	533.9
	contribution [%]	84.1	4.9	9.9	1.2
Au-Beta(IE)	BE [eV]	533.6	531. 9	532.4	534.7
	contribution [%]	77.9	6.7	12.3	3.1
Au-HBeta(IM)	BE [eV]	533.5	531.4	532.1	534.1
	contribution [%]	77.4	4.2	15.3	3.1
Au-HBeta(IE)	BE [eV]	533.5	531.6	532.4	534.5
	contribution [%]	75.4	5.1	17.5	2.0
Au-HBeta(AP)	BE [eV]	533.4	531.4	532.2	534.7
	contribution [%]	86.8	4.9	7.5	0.9
Au-HBeta(DR)	BE [eV]	533.2	531.3	532.1	534.2
	contribution [%]	77.8	4.0	16.9	1.3

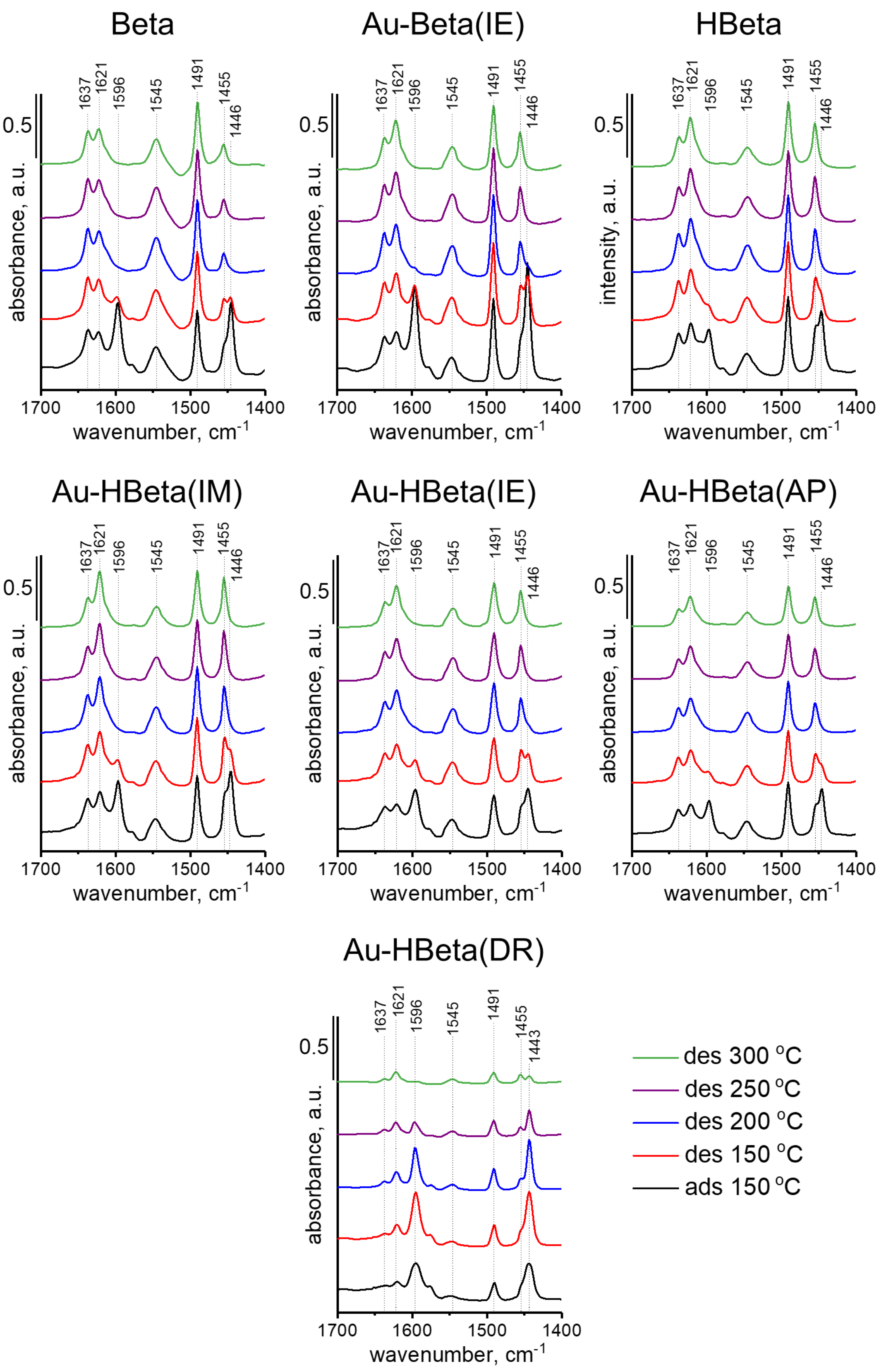

Figure S6. Pyridine ring vibration range of FTIR spectra recorded after adsorption (at 150°C), and desorption of pyridine (at 150, 200, 250, and 300°C). All the spectra were obtained by subtraction the spectrum after activation and normalized to the density of a wafer of 10 mg·cm^{-2}.

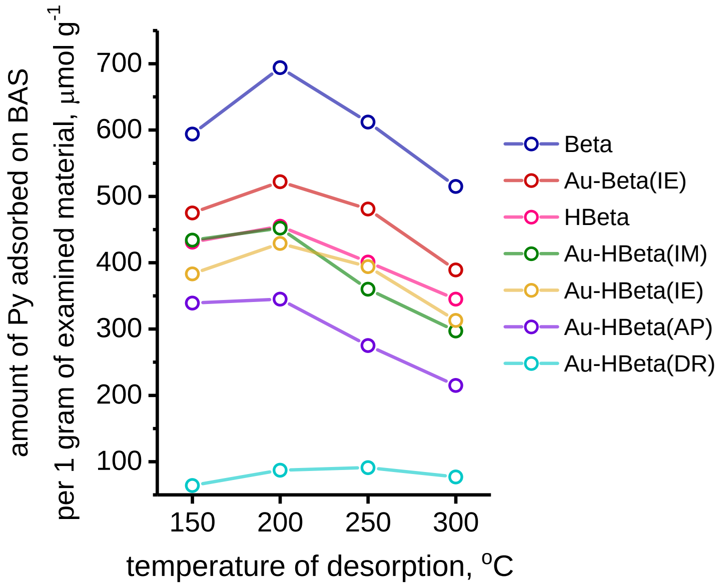

Figure S7. Variations of the amount of pyridine (Py) adsorbed on BAS in relation to evacuation temperature (150, 200, 250, and 300 °C). The calculation based on intensity of 1545 cm^{-1} IR band and extinction coefficient of 0.044 cm^2 μmol^{-1} (from [4]).

References:

1. Amendola, V.; Meneghetti, M. Size evaluation of gold nanoparticles by UV−vis spectroscopy. *J. Phys. Chem. C* **2009**, *113*, 4277–4285, doi:10.1021/jp8082425.
2. Huang, X.; El-Sayed, M.A. Gold nanoparticles: Optical properties and implementations in cancer diagnosis and photothermal therapy. *J. Adv. Res.* **2010**, *1*, 13–28, doi:10.1016/j.jare.2010.02.002.
3. Chaves, T.F.; Pastore, H.O.; Hammer, P.; Cardoso, D. As-synthesized TEA-BEA zeolite: Effect of Si/Al ratio on the Knoevenagel condensation. *Microporous Mesoporous Mater.* **2015**, *202*, 198–207, doi:10.1016/j.micromeso.2014.09.058.
4. Gil, B.; Marszałek, B.; Micek-Ilnicka, A.; Olejniczak, Z. The influence of Si/Al ratio on the distribution of OH groups in zeolites with MWW topology. *Top. Catal.* **2010**, *53*, 1340–1348, doi:10.1007/s11244-010-9592-7.
